# A Prospective Randomized Trial to Compare Safety, Acceptability and Efficacy of Thermal Ablation and Cryotherapy in a Screen and Treat Setting

**DOI:** 10.31557/APJCP.2020.21.5.1391

**Published:** 2020-05

**Authors:** Dipanwita Banerjee, Ranajit Mandal, Amit Mandal, Ishita Ghosh, Srabani Mittal, Richard Muwonge, Eric Lucas, Partha Basu

**Affiliations:** 1 *Chittaranjan National Cancer Institute, Kolkata, West Bengal, India. *; 2 *International Agency for Research on Cancer, Lyon, France. *

**Keywords:** Thermal ablation, cryotherapy, randomized trial, safety, efficacy, acceptability

## Abstract

**Background:**

The prospective randomized study aimed to compare the safety, acceptability and efficacy of thermal ablation (TA) to that of cryotherapy in screen and treat setting.

**Methods:**

The participants were recruited prospectively in a community-based screening clinic in India. Women positive on visual inspection with acetic acid (VIA) test and/or Human Papillomavirus (HPV) test were assessed for eligibility for ablative treatment. Total 286 eligible women were randomized to receive either cryotherapy (N=150) or TA (N=136) performed by health workers. Colposcopy and cervical biopsy were performed on all, prior to treatment. Post-treatment follow-up was after one year with colposcopy and biopsy.

**Results:**

Both the treatment methods had high acceptability. Significantly higher proportion of women treated by cryotherapy reported pain compared to women treated by TA, though intensity was mild in vast majority of them. Approximately 30% of women in both arms had histologic abnormalities, mainly CIN 1, and among those who attended follow-up 74.1% and 81.0% didn’t have any CIN after cryotherapy and TA respectively.

**Conclusion:**

TA is as acceptable and safe as cryotherapy in screen and treat setting. TA has the logistic advantages for the low-resourced settings as the machines are more portable, do not require costly refrigerant gas and battery-driven models are available. The cure rates for CIN 1+ lesions in our study were comparable between cryotherapy and TA.

## Introduction

Screening for cervical cancer followed by treatment of the screen detected abnormalities in the primary care settings has been endorsed by the World Health Organization (WHO) as one of the ‘best buys’ for cancer prevention (WHO, 2017). Over the last decade there has been a paradigm shift in the approach to cervical cancer screening with increasing reliance on screening tests alternative to cytology (e.g. visual inspection with acetic acid (VIA), Human Papillomavirus (HPV) test) and the wider acceptance of alternative management strategies (e.g. screen and treat) (Basu et al., 2017). Ablative treatment of the screen-positive women without disease verification by complex diagnostic tests like colposcopy and histopathology, is not only effective but also safe and well-accepted in low-resourced settings (Gaffikin et al., 2003; Denny et al., 2005; Denny et al., 2010). The guidelines published by the WHO in 2013 recommended cryotherapy as the ablative technique of choice especially in screen and treat settings (WHO, 2013). However, the implementation challenges of cryotherapy in the low and middle income countries (LMICs) are well recognized (Paul et al., 2013; Castle et al., 2017). Only recently the WHO recommended thermal ablation to be used as an alternative to cryotherapy in screen and treat settings (WHO, 2019). However, it was recognized that there was ‘very low certainty in the evidence of effects’ due to the paucity of randomized studies evaluating the safety and efficacy of thermal ablation.

We conducted a randomized study to compare the safety, acceptability and efficacy of TA to that of cryotherapy to treat women positive on VIA and/or HPV detection tests. 

## Materials and Methods

The prospective randomized study was conducted at Chittaranjan National Cancer Institute (CNCI), India and the participants were recruited between February 2016 and July 2017. The study was approved by the CNCI Research Ethics Committee and was registered with Indian Council for Medical Research Clinical Trials Registry (CTRI/2017/06/008731). 

*Selection of participants*

Non-pregnant and previously unscreened women aged between 30 and 60 years were screened in rural community-based clinics with HPV test and VIA. In India there is no population-based screening programme. The screening was purely opportunistic targeting the rural and peri-urban population around the city of Kolkata in Eastern India. The screening was funded by CNCI through a special grant from the Ministry of Health, Government of India. Trained female health workers (N=5) performed speculum examination, collected the cervical specimens for HPV test and then performed VIA. The women positive on VIA were immediately assessed by the health workers to determine their eligibility for ablative treatment based on the following criteria:

• Type I transformation zone (TZ) 

• Lesion does not cover more than 75% of the cervix

• Lesion does not extend to the cervical canal or vagina 

• Lesion can be covered with 25 mm cryo-tip 

• There is no suspicion of cancer 

The cervical specimens were tested for 13 high-risk HPV types by the Hybrid Capture II (HC II, Qiagen, Gaithersburg, Maryland) technology. The women positive on HCII but negative on VIA were recalled at the community clinics (within four weeks of initial visit). The health workers reassessed their eligibility for ablative treatment using the same criteria mentioned earlier. 

Screen-positive women ineligible for ablation were referred to colposcopy and further management based on the colposcopy and biopsy reports.

*Informed consent procedure*

The women eligible for ablative treatment were counselled by a social worker and were invited to participate in the study. While the VIA-positive women could be counselled immediately after VIA, counselling of the HC II positive (and VIA negative) women was done only when they returned for further assessment. Every woman willing to participate signed an informed consent. 

*Randomization procedure*

The randomization to either cryotherapy or TA treatment was done using a computer-generated randomization schedule in batches of 100 to maintain 1:1 distribution between two groups. The treatment assignment was kept in sealed envelopes at trial-office. After completing the consent process, the social worker called the trial-office to obtain the randomization number and corresponding treatment assignment. The assigned treatment was communicated to the health worker who would then perform the procedure. 

*Further assessment by colposcopy*

Every participant had colposcopy by a trained gynecologist immediately after randomization. The colposcopist obtained a punch biopsy from any visible lesion. In absence of any visible lesion, the gynecologist took a punch biopsy from the anterior lip of cervix closest to the squamo-columnar junction. The findings of colposcopy did not alter the treatment decision made by the health worker. All the biopsy specimens were processed and interpreted at the Department of Pathology at CNCI and the results were made available after treatment completion. 

*Treatment procedures*

A health worker performed treatment at the community clinics as per the randomization assignment, immediately after colposcopy. Pregnancy was excluded prior to treatment. The health workers explained the treatment procedure to the women, informed about possible discomfort or pain during treatment and advised to indicate if the pain was too much to continue with the treatment. Cryotherapy was performed using nitrous oxide gas and the double-freeze technique. The health worker reassessed the size of the TZ after applying Lugol’s iodine. She selected a cryo-probe with a tip (slightly conical without nipple) large enough to cover the entire TZ and the lesion. Freezing was done for three minutes followed by five minutes of thawing and three minutes of repeat freezing. Satisfactoriness of treatment was assessed by observing the formation of an ice-ball extending a few millimeters beyond the rim of the probe-tip. 

TA was performed using the cold-coagulator (WISAP^®^ Medical Technology GmbH) after delineating the TZ with Lugol’s iodine. The 20 mm flat probe was heated to 100o C and was applied to the cervix for 40 seconds. Multiple overlapping applications with same probe were made to cover the TZ/lesion adequately. 

The cervix and vagina were examined before withdrawal of the speculum for any bleeding or accidental injury. No anesthesia or pre-operative analgesics were used for either procedure.

*Post-treatment care*

Every woman was advised to lie down for 5-10 minutes after the procedure. The health worker observed the woman for any side-effects like pain, abdominal cramps, bleeding or vasovagal reactions. The woman was then allowed to get up and see a clinician for advice. The clinician gave instructions on self-care, the symptoms to expect and follow-up visits. The women were informed about the watery vaginal discharge that they could expect for one to two weeks. They were advised to avoid sexual intercourse for one month. If abstinence was not feasible, they were advised to ensure that their partners used condom during sex. The women were advised to report back to the treatment center or call the social worker if they suffered excessive pain, vaginal bleeding exceeding normal menstruation with passing of blood clots and foul-smelling vaginal discharge with fever. No prophylactic antibiotics were prescribed. Every woman was advised follow-up after 9-12 months.

*Assessment of pain intensity and level of satisfaction*

Before sending the woman home the clinician asked her to score the intensity of pain experienced during the procedure on a visual rating scale with values ranging from 1 (‘no pain at all’) to 9 (‘so much pain that you wanted the procedure to be stopped’). The woman was also asked to rate her level of satisfaction with the screening and treatment services using another visual rating scale with scores ranging from 1 (‘not satisfied at all’) to 9 (‘highly satisfied’). 

*Follow up assessment*

Follow up was planned at 1 year after treatment. All the women underwent colposcopy at follow-up and had biopsies directed from the lesions detected on colposcopy. A punch biopsy was obtained from the anterior lip of cervix, if no lesion was visible. The colposcopists and the pathologists were blinded to the treatment allocation. 

*Statistical considerations*

Descriptive analyses were presented using proportions for categorical variables and mean together with standard deviation (SD) or median together with the interquartile range (IQTR) for the continuous variables. Comparisons of the categorical variables were done using the Pearson’s chi-square test. An ordered logistic regression model was used to assess the effect of multiple applications on the pain levels of the patients during thermal ablation treatment. Because of the scarcity of data in some the cells of the pain level-number of application cross-tabulation, the true proportions in these cells were then estimated from the regression model. Cure rates were estimated only for the women showing CIN 1 or worse (CIN 1+) lesions on baseline histopathology. The data was analyzed using the STATA software, version 14.0 (StataCorp, College Station, Texas, US). The sample size was decided empirically.

## Results

**Figure 1:**
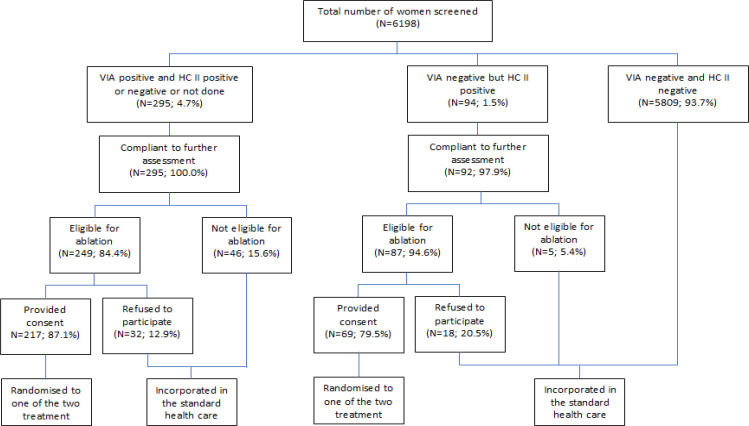
Selection Process for Randomization

Total 286 women were randomized to receive either cryotherapy (N=150) or TA (N=136). The selection of 286 eligible women out of the total 6198 women screened is described in figure 1. The mean age was 36.1 (SD: 5.6) years in cryotherapy and 36.7 (SD: 7.3) years in TA arm. The randomized arms were well matched for age, various reproductive and socio-demographic factors at enrolment (Table 1). 

The outcomes of the screening tests, colposcopy and histopathology at baseline are shown in table 2. In the cryotherapy arm 78.7% (118/150) were VIA-positive at baseline. Additional 32 women (21.3%) were recruited in the cryotherapy arm as they were positive on HCII test (though VIA was negative) at baseline. The corresponding figures for the TA arm were 72.8% (99/136) and 27.2% (37/136), which were not significantly different. Overall 33.6% (40/119) of the women recruited to cryotherapy arm and 43.4% (49/113) of those recruited to the TA arm were positive for high risk HPV. Some of the women did not have HPV test. Among the VIA positive and HCII negative women 15.6% (46/295) were ineligible for ablative treatment as they did not fulfil the criteria mentioned earlier. The corresponding rate in the HCII positive and VIA negative women was 5.4% (5/92). The ineligible women were referred for excisional treatment.

In both study arms, nearly half of the VIA-positive women had the acetowhite area occupying <25% of the cervix and the rest half had lesions occupying 25-50%. None of the randomized women were excluded due to colposcopic suspicion of cancer. Baseline histopathology revealed CIN 1 in 27.7% (41/150) women recruited to the cryotherapy arm and 29.6% (40/136) women in the TA arm. Histopathology proved high grade lesions (CIN 2/CIN 3) were detected in 5 women each in the cryotherapy (3.3%) and the TA (3.7%) arms. There was no statistically significant difference in histopathology distribution between the two arms.

Treatment could be completed in 100% of the randomized women. All women in the cryotherapy arm were treated with double-freeze technique. In the TA arm majority (61.8%) were treated with a single application of the probe. Two, three and four applications were required in 33.1%, 4.4% and 0.7% women respectively. Pain or cramp in the lower abdomen during or immediately after the procedure was the most frequent side-effect and was reported by significantly higher proportion of women in the cryotherapy arm (75.3%) compared to the TA arm (61.0%) (Table 3). The other side effects were negligible.

The comparison of the intensity of pain during or immediately after the procedure indicated by the participants using the visual scale is shown in table 3. The fact noted by the treatment provider that a significantly higher proportion of the women experienced pain (though mostly mild in intensity) in the cryotherapy arm was also corroborated by the objective pain assessment by the patients. Very few women felt severe pain (scored 7 to 9) during either cryotherapy (1.3%) or TA (1.5%). The true proportions (estimated from the ordered logistic regression model) of moderate and severe pain intensity increased with increasing number of treatment applications in the TA arm (Table 3). Almost all women in both the study arms indicated high level of satisfaction and agreed to recommend screening to their friends and family members. None of the treated women reported any major complication post-treatment, like excessive bleeding or pelvic inflammatory disease. 

**Figure 2:**
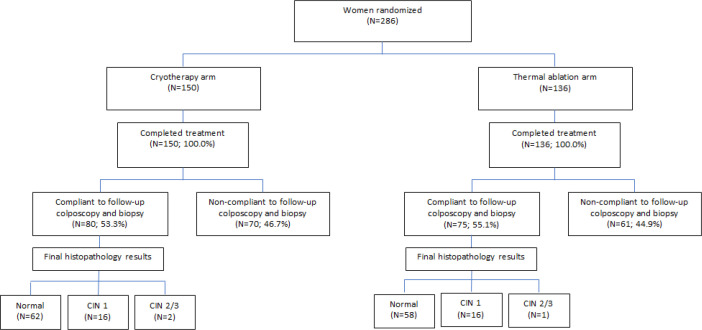
Treatment and Follow-up Outcomes

The follow-up compliance was low in both the study arms; 53.3% (80/150) in the cryotherapy arm and 51.5% (70/136) in the TA arm. In the cryotherapy arm 56.3% (27/48) women with a baseline diagnosis of CIN 1+ underwent follow-up colposcopy. The corresponding figure in the TA arm was 45.7% (21/46). The follow-up outcomes are described in figure 2 and table 4. The median follow-up times were 11.6 (IQR: 9.4-13.0) months for the cryotherapy arm and 11.2 (IQR: 8.9-13.8) months for the TA arm. Among the 80 women treated with cryotherapy and successfully followed up, 16 cases of CIN 1 and 2 cases of CIN 2/3 were detected on final histopathology (Figure 2). Out of the 75 women treated by cryotherapy and followed up, 16 cases on CIN 1 and one case of CIN 2/3 were detected on follow up biopsy. Some these CIN 1+ lesions were new in the women who didn’t show any CIN on baseline histopathology. Only the women showing CIN 1+ lesions on baseline histopathology were included in the assessment of cure. The cure rates of CIN 1+ lesions were 74.1% (20/27) and 81.0% (17/21) in the cryotherapy and TA arms respectively (p=0.57). The cure rates of CIN 1+ among the women positive for HPV at baseline were 100.0% (6/6) and 81.8% (9/11) in the cryotherapy and TA arms respectively. Only two women with CIN 2/3 at baseline and treated by cryotherapy underwent follow up; one had CIN 1 and the other was normal. Both the women with CIN 2/3 at baseline undergoing follow up after TA were free of any CIN. 

**Table 1:** Participants Baseline Characteristics by Randomization group

Baseline characteristics	Randomization group		Chi^2^ *p*-value
	Cryotherapy n (%)	Thermal ablation n (%)	
Women assessed	150	136	
Age (years)			
30-39	108 (72.0)	95 (69.9)	0.03
40-49	39 (26.0)	29 (21.3)	
50-60	3 (2.0)	12 (8.8)	
Education			
None	33 (22.0)	35 (25.7)	0.653
Primary	76 (50.7)	59 (43.4)	
Secondary	37 (24.7)	37 (27.2)	
College/University	4 (2.7)	5 (3.7)	
Occupation			
House wife	142 (94.7)	127 (93.4)	0.67
Manual	4 (2.7)	6 (4.4)	
Office going	3 (2.0)	3 (2.2)	
Other	1 (0.7)	0 (0.0)	
Marital status			
Unmarried	3 (2.0)	3 (2.2)	0.852
Married/cohabiting	144 (96.0)	128 (94.1)	
Widowed	2 (1.3)	3 (2.2)	
Separated	1 (0.7)	2 (1.5)	
Type of house			
Thatched	39 (26.0)	32 (23.5)	0.882
Tiled	82 (54.7)	76 (55.9)	
Concrete	29 (19.3)	28 (20.6)	
Residence area			
Urban	10 (6.7)	13 (9.6)	0.638
Semi-urban	6 (4.0)	5 (3.7)	
Rural	133 (88.7)	118 (86.8)	
Unknown	1 (0.7)	0	
Age at marriage (years)			
<15	10 (6.7)	11 (8.1)	0.8
15-18	97 (64.7)	90 (66.2)	
18+	43 (28.7)	35 (25.7)	
Total pregnancies			
0-1	18 (12.0)	9 (6.6)	0.289
2-3	98 (65.3)	96 (70.6)	
4+	34 (22.7)	31 (22.8)	
Last menstruation			
<1 year	142 (94.7)	123 (90.4)	0.171
>1 year	8 (5.3)	13 (9.6)	

**Table 2:** Baseline Clinical and Pathological Findings by Randomization Group

	All participants	Randomization group		Chi^2^ *p*-value
		Cryotherapy n (%)	Thermal ablation n (%)	
VIA findings				
Negative	69 (24.1)	32 (21.3)	37 (27.2)	0.246
Positive	217 (75.9)	118 (78.7)	99 (72.8)	
Total	286 (100.0)	150 (100.0)	136 (100.0)	
If positive, size of the acetowhite area				
<25%	107 (49.3)	55 (46.6)	52 (52.5)	0.352
25%-50%	109 (50.2)	63 (53.4)	46 (46.5)	
50%-75%	1 (0.5)	0 (0.0)	1 (1.0)	
Total	217 (100.0)	118 (100.0)	99 (100.0)	
HC2 test (if performed)				
Negative	143 (61.6)	79 (66.4)	64 (56.6)	0.127
Positive	89 (38.4)	40 (33.6)	49 (43.4)	
Total	232 (100.0)	119 (100.0)	113 (100.0)	
Colposcopy diagnosis				
Normal	116 (40.7)	61 (40.7)	55 (40.7)	0.978
Probable CIN 1	155 (54.4)	82 (54.7)	73 (54.1)	
Probable CIN 2/3	14 (4.9)	7 (4.7)	7 (5.2)	
Total	285 (100.0)	150 (100.0)	135 (100.0)	
Histopathology report				
Normal	192 (67.8)	102 (68.9)	90 (66.7)	0.904
CIN 1	81 (28.6)	41 (27.7)	40 (29.6)	
CIN 2	5 (1.8)	3 (2.0)	2 (1.5)	
CIN 3	5 (1.8)	2 (1.4)	3 (2.2)	
Total	283 (100.0)	148 (100.0)	135 (100.0)	
Final diagnosis				
Normal	192 (67.1)	102 (68.0)	90 (66.2)	0.916
CIN 1	84 (29.4)	43 (28.7)	41 (30.1)	
CIN 2	5 (1.7)	3 (2.0)	2 (1.5)	
CIN 3	5 (1.7)	2 (1.3)	3 (2.2)	
Total	286 (100.0)	150 (100.0)	136 (100.0)	
Reference standard				
Colposcopy	3 (1.0)	2 (1.3)	1 (0.7)	0.62
Histopathology	283 (99.0)	148 (98.7)	135 (99.3)	
Total	286 (100.0)	150 (100.0)	136 (100.0)	

VIA, visual inspection with acetic acid; HC2, hybrid capture 2; CIN, cervical intraepithelial neoplasia

**Table 3:** Number of Applications, Side-Effects, Pain or Discomfort Levels During Treatment, Satisfaction Levels after Treatment and Screening Recommendation to Others

	Randomization group		Chi^2^ *p*-value
	Cryotherapy	Thermal ablation	
	n (%)	n (%)	
Women assessed	150	136	
Number of applications			
One	0	84 (61.8)	
Two	150 (100.0)	45 (33.1)	
Three	0	6 (4.4)	
Four	0	1 (0.7)	
Side-effects during procedure (acceptability)			
None	35 (23.3)	53 (39.0)	0.022
Pain/cramps	113 (75.3)	83 (61.0)	
Bleeding	1 (0.7)	0 (0.0)	
Vasovagal reaction	1 (0.7)	0 (0.0)	
Intensity of pain or discomfort during/immediately after the procedure			
1-3	134 (89.3)	131 (96.3)	0.039
4-6	14 (9.3)	3 (2.2)	
7-9	2 (1.3)	2 (1.5)	
Level of satisfaction with services			
1-3	0 (0.0)	1 (0.7)	0.147
4-6	3 9 (2.0)	0 (0.0)	
7-9	147 (98.0)	135 (99.3)	
Woman to recommend the screening procedure to others			
Yes	149 (99.3)	136 (100.0)	0.34
No	1 (0.7)	0	

**Table 4:** Cure Rates after Baseline CIN Treatment*

	Baseline CIN									
	CIN 1		CIN 2		CIN 3		CIN 2/3		All CIN	
	CT	TA	CT	TA	CT	TA	CT	TA	CT	TA
VIA &/or HPV positive women having CIN on histopathology										
Women treated at baseline	43	41	3	2	2	3	5	5	48	46
Women followed up after treatment, n	25	19	2	1	0	1	2	2	27	21
%	58.1	46.3	66.7	50	0	33.3	40	40	56.3	45.7
Women with no evidence of disease at follow-up, n	19	15	1	1		1	1	2	20	17
%	76	78.9	50	100		100	50	100	74.1	81
Women diagnosed with CIN at follow-up, n										
CIN 1	5	4	1	0		0	1	0	6	4
CIN 2	0	0	0	0		0	0	0	0	0
CIN 3	1	0	0	0		0	0	0	1	0
HPV positive women having CIN on histopathology										
Women treated at baseline	8	13	1	2	2	3	3	5	11	18
Women followed up after treatment, n	5	9	1	1	0	1	1	2	6	11
%	62.5	69.2	100	50	0	33.3	33.3	40	54.5	61.1
Women with no evidence of disease at follow-up, n	5	7	1	1		1	1	2	6	9
%	100	77.8	100	100		100	100	100	100	81.8
Women diagnosed with CIN at follow-up, n										
CIN 1	0	2	0	0		0	0	0	0	2
CIN 2	0	0	0	0		0	0	0	0	0
CIN 3	0	0	0	0		0	0	0	0	0

*Includes only the cases with CIN 1 – CIN 3 on baseline histopathology; CT, cryotherapy group; TA, thermal ablation group; CIN, cervical intraepithelial neoplasia; HPV, human papilloma virus

## Discussion

Our study corroborates the findings of another recently published RCT from Zambia that also demonstrates TA to be as safe and as efficacious as cryotherapy and very well accepted by the women in a screen and treat setting (Pinder et al., 2020). While the study by Pinder et al (2020) could not verify the disease status histopathologically, either at baseline or at follow up, we have information on histopathology on all participants at both time points. 

Even though WHO recently recommended TA as a safe and efficacious alternative to cryotherapy to treat CIN 2/3 lesions, such recommendation was based on weak evidence. A good number of observational studies and only one RCT by Singh et al., (1988) have demonstrated the high efficacy of TA to treat histopathology proved cervical neoplasias in various settings and even in the HIV infected women (Hussein and Galloway, 1985; Smart et al., 1987; Gordon and Duncan, 1991; Joshi et al., 2013; Campbell et al., 2016; Naud et al., 2016). However, several meta-analysis and systematic reviews of such studies underscored the paucity of information related to the adverse events during and after the procedure (Dolman et al., 2014; Cremer et al., 2018; Randall et al., 2019). Concerns have been expressed by some authors regarding the possibility of the women feeling more pain during TA (Maza et al., 2016). The intensity of pain during TA has been reported only by Hussein and Galloway (1985), Joshi et al., (2013), Naud et al., (2016) and Pinder et al., (2020). The low occurrence of moderate to severe pain reported in these studies matches with ours (Hussein and Galloway, 1985; Joshi et al, 2013; Naud et al, 2016; Pinder et al, 2020). The earlier studies, except the one by Pinder et al., (2020) reported only the subjective assessment by the treatment providers, which might have influenced the outcomes. In our study we documented the intensity of pain perceived by the patients in a more objective manner along with the independent pain assessment by the providers. To minimize the social acceptability bias, the patients reported the pain intensity to a clinician not involved in treatment. Very few patients experienced severe pain during either of the ablative procedures and the proportion of women reporting mild to moderate pain was actually higher in the cryotherapy arm. The difference may be because of few seconds of treatment time with TA compared to at least 12 minutes for cryotherapy. The pain intensity increases with increasing number of applications of TA probe. We have also demonstrated that TA is as acceptable to the women as cryotherapy and the women have high level of satisfaction after treatment by either of the procedures. 

The major limitations of our study are the low number of high grade lesions in the screen positive women and the poor follow up rate. Earlier large community-based studies in the same population in eastern India demonstrated the low prevalence of cervical precancers and cancers. One such study by Basu et al involving nearly 40,000 women aged 30 to 60 years observed a CIN 2/CIN 3 detection rate of less than 5/1,000 women screened with VIA and HC II (Basu et al., 2015). The low prevalence of disease led to a low positive predictive value (PPV) of 6.9% and 10.8% to detect CIN 2 or worse lesion for VIA and HCII respectively. In the current study the PPV of the screening test is even lower as only those with smaller ectocervical lesions or no visible lesions were eligible for ablative treatment. The low number of CIN 2/CIN 3 lesions has made our study underpowered to compare the cure rates against histology-proved CIN 2 and CIN 3. We observed a similar rate of cure of CIN 1+ lesions in the treatment arms. However, majority of the lesions was CIN 1, which is a self-limiting disease and is not a valid efficacy endpoint. A study with higher sample size is required to establish the efficacy against more valid endpoints.

On the other hand, our study reveals the high rate of over-treatment in a screen and treat approach, particularly in settings with low to moderate prevalence of disease. Overall 67.1% of the women treated did not have any CIN lesion. Even in a high disease prevalent setting in Zambia (due to high prevalence of HIV infection) Pinder et al., (2020) observed that nearly half of the women treated did not have any cervical neoplasias.

In conclusion, our study demonstrates TA to be safe and effective in a screen and treat setting, even if the procedure is performed by health workers. A randomized study with adequate sample size is needed to conclusively demonstrate efficacy of TA to treat CIN 2/CIN 3 lesions. A significant rate of over-treatment is inevitable in ‘screen and treat’ approach. Considering the challenges in ensuring regular follow ups in the LMICs as shown in our study, the benefits of over-treatment with a safe, simple, acceptable and affordable technique like TA outweigh the risks. 
